# Early Psychosis Intervention Programme in a Healthcare Area of Málaga: a descriptive study

**DOI:** 10.1192/j.eurpsy.2022.1966

**Published:** 2022-09-01

**Authors:** E. Sánchez Martínez, C. Gómez Sanchez-Lafuente, A.B. Mulero Garcia

**Affiliations:** Hospital Regional Universitario de Málaga, Ugc Salud Mental, Málaga, Spain

**Keywords:** programme, First-episode psychosis, early psychosis intervention programme, Psychosis

## Abstract

**Introduction:**

The first years after a first-episode psychosis are crucial, and a comprehensive approach is essential.

**Objectives:**

Our objective is to observe the characteristics of patients attending Early Psychosis Intervention Programme in (PITP) of the Mental Health Clinical Management Unit (UGC) of the Hospital Regional Universitario de Málaga between the years 2016-2020.

**Methods:**

Data for this retrospective study were derived from 135 patients included in the PITP database between the years 2016-2020 from the Mental Health UGC of the Hospital Regional Universitario de Málaga. Descriptive analysis was performed using SPSS 25.0. For the comparison of variables, Student’s t was used for quantitative variables and Chi square for dichotomous ones.

**Results:**

68% of the patients were men, compared to 32% women. The mean age of the patients at the beginning of the Programme was 30.76 years. 85% were 40 years or younger. 67.4% lived with their family of origin. 58.5% of the patients have had at least one hospitalization in Psychiatry. 45.9% of the patients were smokers, 33.3% consumed alcohol and 59.3% consumed other drugs. From the patients who started the Programme, 38.5% continue with the follow-up, 22.2% were discharged and 39.3% abandoned it. More results will be presented on the poster.

**Conclusions:**

In conclusion, the patients included in the PITP of our UGC present characteristics compatible with the current bibliography. The high percentage of withdrawal from the Programme stands out, this being a very important point to address and improve.

**Disclosure:**

No significant relationships.

